# Lentiviral-Mediated Short Hairpin RNA Knockdown of MTDH Inhibits Cell Growth and Induces Apoptosis by Regulating the PTEN/AKT Pathway in Hepatocellular Carcinoma

**DOI:** 10.3390/ijms160819419

**Published:** 2015-08-17

**Authors:** Wen-Fang Li, Qin Ou, Hang Dai, Chang-An Liu

**Affiliations:** 1 Department of Hepatibiliary Surgery, the Second Affiliated Hospital of Chongqing Medical University, Chongqing 400016, China; E-Mails: wen_fang79@yahoo.com (W.-F.L.); hangdaicq@sina.com (H.D.); 2 Department of Cancer Research Center, Hubei Medical University, Shiyan 442000, China; E-Mail: ouqinzaisy@sina.com; 3 State Key Laboratory of Ultrasound Engineering in Medicine Co-Founded by Chongqing and the Ministry of Science and Technology, Shiyan 442000, China

**Keywords:** hepatocellular carcinoma, growth, apoptosis, metadherin

## Abstract

The activation of oncogenes and the loss of tumor suppressor genes are believed to play critical roles in the pathogenesis of human hepatocellular carcinoma (HCC). Metaherin (MTDH), also called astrocyte elevated gene-1 (AEG-1), is frequently amplified in a variety of cancers, but the roles of MTDH with regard to growth and apoptosis in HCC have not yet been studied. In the present study, we first analyzed the expression of MTDH in HCC samples. We found that MTDH protein levels are higher in most HCC cancerous tissues compared with their matched adjacent non-tumor tissues. Additionally, the MTDH mRNA was also higher in HCC tissues compared to their matched adjacent non-tumor tissues. Knockdown of the endogenous MTDH using small interfering RNA further showed that deficiency of MTDH suppressed cell growth and caused apoptosis in HCC cells. Knockdown MTDH promoted PTEN and *p53* expression in HCC cells and inhibited AKT phosphorylation. Knockdown MTDH also inhibited tumor growth *in vivo*. All these results indicated that MTDH protein levels in most HCC tissues are higher than non-tumor tissues, and knockdown of MTDH inhibited growth and induced apoptosis in HCC cells through the activation of PTEN. Therefore, MTDH might be an effective targeted therapy gene for HCC.

## 1. Introduction

Hepatocarcinogenesis is a complex multistep process in which many signalling cascades are altered, leading to a heterogeneous molecular profile. Comprehensive gene mutations and abnormal gene/protein expression are intimately correlated with hepatocellular cancer generation [1]. Surgery remains the main therapy for HCC, but only 30% had the opportunity to surgically remove the tumor, and the postoperative five-year survival rate was only about 30%–40% for liver cancer patients [2]. Moreover, liver transplantation is a fundamental treatment method, but liver sources are very scarce [3]. Gene therapy has been emerging as a promising intervention against HCC. However, due to the complexity of signaling pathways that initiate and maintain the occurrence and progression of HCC, the poor understanding of underlying molecular mechanisms in HCC development impede HCC therapy. The identification of a new target gene that is effective and specific for HCC malignant behavior is urgently required to improve HCC therapy. Also, the discovery of oncogenes associated with HCC growth and clarifying their mechanism might provide important clues for HCC clinical treatment [4].

MTDH is a single-pass transmembrane protein composed of 582 amino acids with a gene located at chromosome 8q22.5 [5]. Overexpression of MTDH is frequently observed in melanoma, breast cancer, prostate cancer, and esophageal cancer and is correlated with poor clinical outcomes [6]. Many signaling pathways are regulated by MTDH, such as nuclear factor-kappa B, Wnt/β-catenin, MAPK/ERK, and PI3K/AKT. In HCC patients, it was also found that the clinical outcome was consistently poorer for the high MTDH expression group than for the low MTDH expression group [7,8]. High MTDH expression in HCC is positively correlated with tumor microvascular invasion, tumor grade and stage, and high recurrence rate [9]. Also, deletion of MTDH could effectively inhibit hepatocarcinogenesis [10,11]. However, the molecular mechanisms of MTDH promoting HCC growth have not been fully investigated.

In the present study, we aimed to examine the effects of MTDH silencing on HCC cell viability *in vitro* and tumor growth *in vivo*. Additionally, we evaluated the cell apoptosis in MTDH knockdown HepG2 cells. Finally, we tested the proliferation and apoptosis-related protein expression changes in MTDH shRNA HepG2 cells, in an attempt to explore the molecular mechanisms in MTDH mediating HCC growth.

## 2. Results

### 2.1. Expression of MTDH Was Significantly Upregulated in HCC Tissues

**Figure 1:**
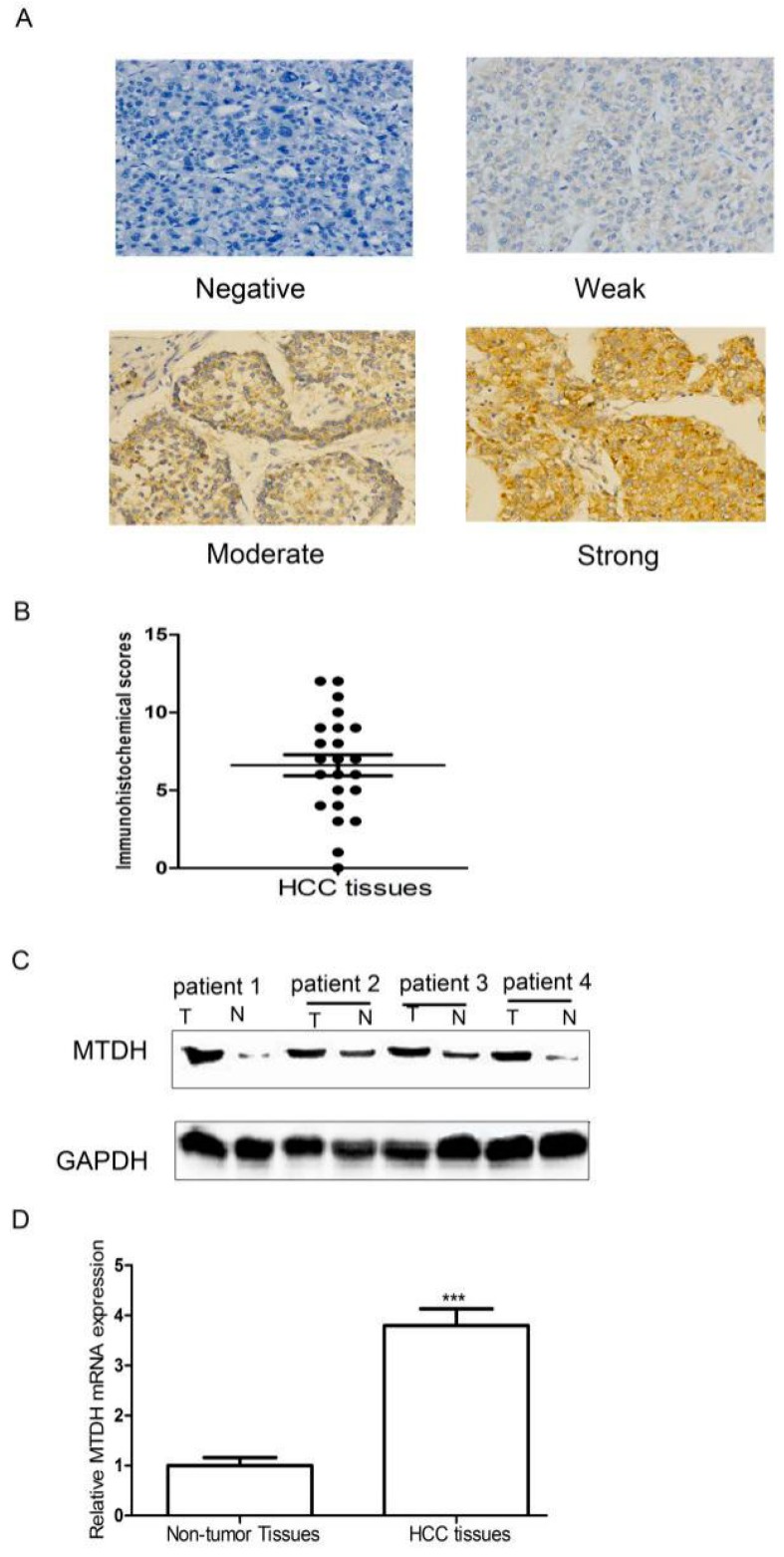
Expression situation of MTDH. (**A**) Representative MTDH microphotographs of immunohistochemistry (200×); (**B**) Representative immunohistochemical scores of MTDH in HCC tissues; (**C**) Western blot was used to compare MTDH protein expression in HCC tissues and matched noncancerous liver tissues. (T: tumor tissues and N: non-cancerous liver tissues); (**D**) q-PCR to test MTDH mRNA expression in HCC tissues and matched noncancerous liver tissues*.* *** *p* < 0.01

To investigate the roles of MTDH in HCC progression, we first used immunohistochemistry to test MTDH in HCC tissues. The results showed that MTDH was differently expressed in HCC tissues. The positive expression of MTDH in HCC tissues was 65.22% (15/23). MTDH expression was mainly located in the cytoplasm and cell membrane, and MTDH was also found in the nucleus in HCC tissues (Figure 1A). The immunohistochemical scores of MTDH in HCC tissues are shown in Figure 1B. We also determined the expression level of MTDH in 4 pairs of HCC tissues and matched non-cancerous liver tissues via Western blot. As shown in Figure 1C, higher expression of MTDH in HCC tissues compared with matched non-cancerous liver tissues was found. SYBR green-based qRT-PCR assays were carried out to detect the expression level of MTDH in 12 HCC tissues and matched adjacent non-tumor tissues. We found that MTDH mRNA expression in HCC tissues was higher compared to adjacent matched non-tumor tissues (Figure 1D). Thus, we showed that the both protein and mRNA expression level of MTDH was significantly increased in HCC tissues compared to matched non-cancerous liver tissues. These results suggested that MTDH might have important roles in HCC pathogenesis.

### 2.2. shRNA Silencing of MTDH Expression Effectively Reduces MTDH mRNA and Protein Expression

**Figure 2:**
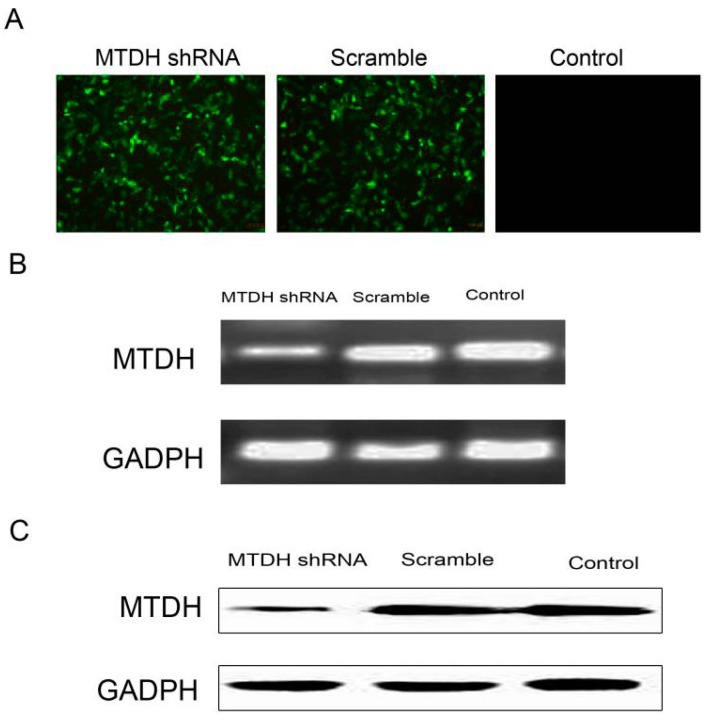
shRNA silencing of MTDH expression effectively reduces MTDH mRNA and protein expression in HepG2 cells. (**A**) Stably transfected HepG2 cells on fluorescence microscopy (200×); (**B**) q-PCR quatification of MTDH mRNA, and products were observed with 1.5% agarose gel containing 0.5 μg/mL ethidium bromide with an ultraviolet illuminator; (**C**) Western blot to test MTDH protein expression.

To further explore the molecular mechanism of MTDH-mediated pathological functions in HCC, we examined knockdown MTDH protein expression in HepG2 cells. Cells were infected by a lentivirus which contained MTDH shRNA (LV-GFP-MTDH-shRNA) or an empty vector (LV-GFP-NC-shRNA) (Figure 2A). Uninfected HepG2 cells were included as the negative control. The infected efficiency was calculated by dividing fluorescent HepG2 cell number and total HepG2 cells with fluorescence microscopy. The infected efficiencies were 93.2% and 92.8% in the LV-GFP-MTDH-shRNA and LV-GFP-NC-shRNA groups, respectively. Following infection, the cells were collected for mRNA and protein expression analysis. As indicated in Figure 2B, apparently, compared with the LV-GFP-NC-shRNA cells and uninfected negative control cells, the mRNA and protein expression levels of MTDH in the LV-GFP-MTDH-shRNA cells was decreased. Based on the RT-PCR results, the mRNA level of MTDH was reduced by 89.6% in the LV-GFP-MTDH-shRNA cells, while the protein level of MTDH was significantly reduced by 73.8% in the LV-GFP-MTDH-shRNA cells *vs*. that in the LV-GFP-NC-shRNA and control cells as detected by Western blot.

### 2.3. Knockdown of MTDH Inhibits the Viability, Colony Formation and Induced Apoptosis in HepG2 Cells

**Figure 3:**
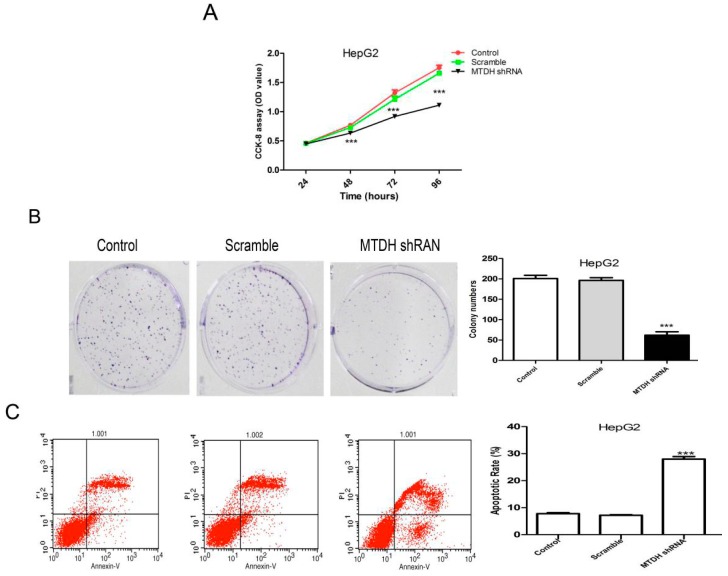
Effects of MTDH silencing on HepG2 cell proliferation and apoptosis. (**A**) CCK-8 to test cell proliferation; (**B**) Representative photographs of the colony formation assay. Quantification of the colony number, *** *p* < 0.001; (**C**) Annexin V–FITC/PI assay for determination of apoptosis of MTDH silencing on HepG2 cells with a flow cytometer. The apoptosis results were analyzed with Student’s *t*-test (*** *p* < 0.001).

To understand whether down-regulation of MTDH inhibits HepG2 cells viability, CCK-8 assay was used to test the viability effect of shRNA silencing of MTDH expression in HepG2 cells. As shown in Figure 3A, shRNA silencing of MTDH expression significantly inhibited HepG2 cell viability at 48, 72 and 96 h compared to negative control group. A colony formation assay also showed that shRNA silencing of MTDH expression could significantly inhibit HepG2 colony formation compared to the negative control group (Figure 3B).

Cell apoptosis is an important cause of viability suppression, so we also performed a cell apoptosis assay with a flow cytometer. The percentage of apoptosis in HepG2 cells was greatly increased in the MTDH shRNA group (Figure 3C). Our results revealed that MTDH had a tumor growth-promoting effect in HCC tumors. This strongly supported the potential finding that anti-cancer therapy via targeting MTDH in HCC might have great value.

### 2.4. Knockdown of MTDH Inhibits Phosphorylation of AKT and Increased Apoptosis Related Protein Expression

**Figure 4:**
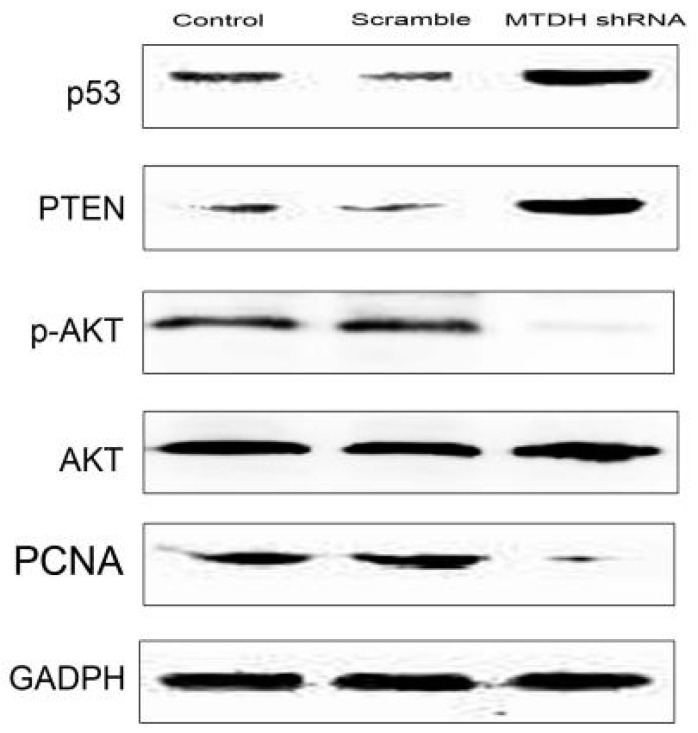
MTDH silencing effects on growth and apoptosis related protein expression. Knockdown MTDH expression in HepG2 cells increased PTEN and p53 expression, while MTDH shRNA could effectively inhibit phosphorylation of AKT and PCNA expression.

PTEN is tightly controlled by various non-genomic mechanisms. To further determine molecular mechanisms of MTDH in HCC growth, we tested the growth- and apoptosis-related protein PTEN expression in HepG2 stable cells with or without shRNA silencing of MTDH expression. As indicated in Figure 4, MTDH shRNA could effectively increase PTEN and p53 expression. MTDH shRNA also inhibited phosphorylation of AKT and thus inhibited AKT activation. The wild type p53 protein was higher compared to LV-GFP-NC-shRNA and control groups. These results suggested that MTDH regulated multiple types of growth- and apoptosis-related protein expression in HCC.

### 2.5. Knockdown of MTDH Inhibits HepG2 Tumor Growth in Xenograft Model

**Figure 5:**
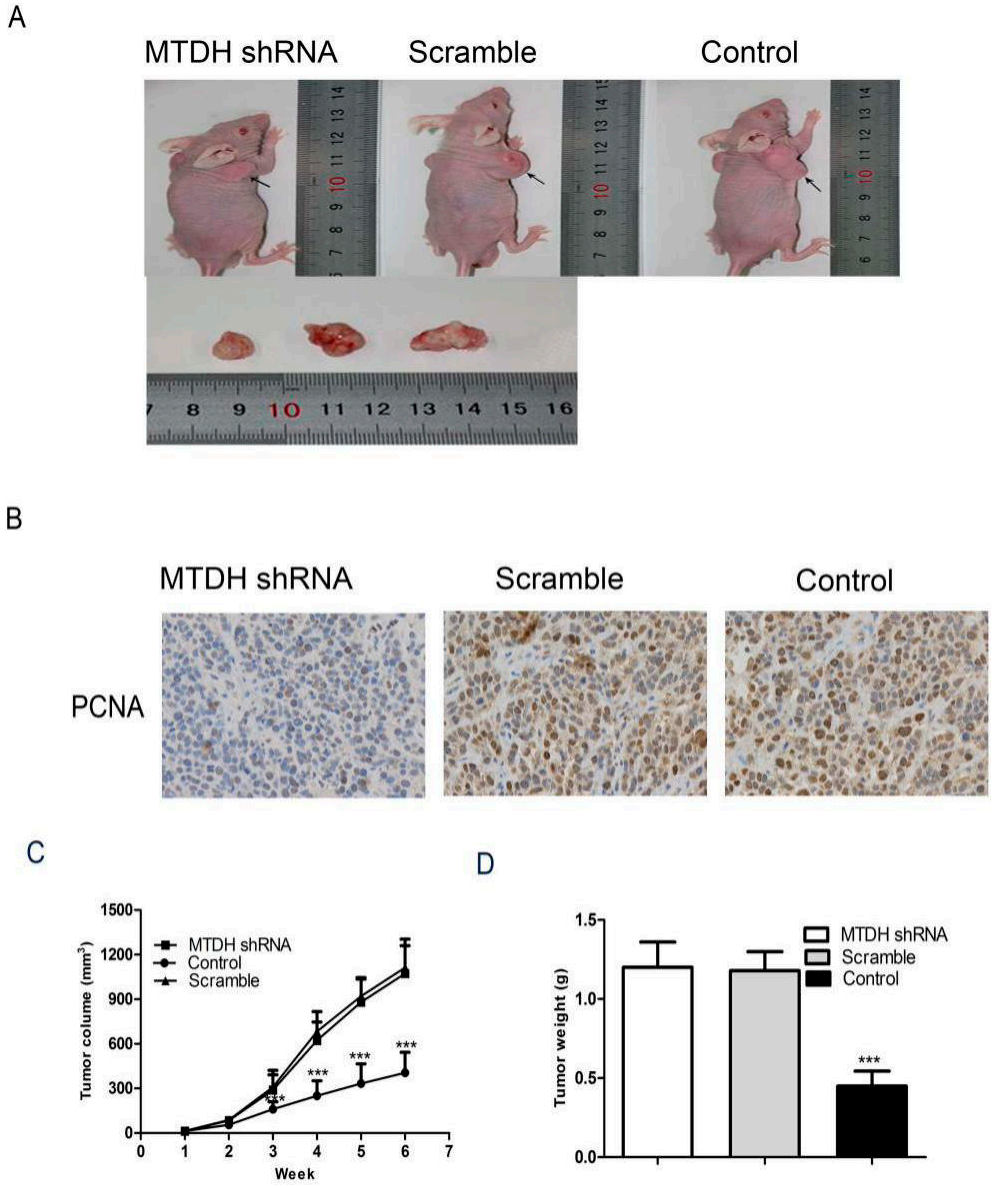
MTDH silencing suppresses HepG2 tumorigenicity *in vivo*. HepG2 cells were infected with LV-GFP-MTDH-shRNA or LV-GFP-NC-shRNA. After infection for 48 h, the cells were collected. (**A**) Photographs of dissected tumors from nude mice; (**B**) PCNA expression was tested with immunohistochemistry (200×); (**C**) tumor growth curves measured after subcutaneous injection of HepG2 cells were infected with LV-GFP-MTDH-shRNA or LV-GFP-NC-shRNA. The tumor volume was calculated for 6 weeks. *** *p* < 0.001, Student’s *t*-test); (**D**) the tumor tissues from the animal were weighed. (*** *p* < 0.001, Student’s *t*-test).

Nude mice was subsequently injected with LV-GFP-MTDH-shRNA or LV-GFP-NC-shRNA cells into the right axilla of BALB/c nude mice. The mice were sacrificed 6 weeks after inoculation and tumors were excised and measured (Figure 5A). The tumor volume of mice bearing LV-GFP-MTDH-shRNA tumors was 38% that of mice bearing LV-GFP-MTDH-shRNA tumors (Figure 5B). And immunohistochemical results showed that LV-GFP-MTDH-shRNA significantly inhibited PCNA expression compared to LV-GFP-NC-shRNA tumors. Furthermore, the weight of LV-GFP-MTDH-shRNA tumors was 36% LV-GFP-NC-shRNA tumors (Figure 5C).

## 3. Discussion

The prognosis for HCC is still very poor due to the high frequency of late-stage cancer and metastasis at diagnosis. Despite benefits from surgical resection and a low risk of complications, only a limited proportion of HCC patients are eligible to undergo optional resection at diagnosis. Chemotherapy represents the main therapeutic option for these HCC patients, but neither single-drug nor multiple-drug treatments prolong the survival of late-stage HCC patients [12]. Knowledge of molecular events that govern tumor progression and dissemination has allowed the development of targeted treatments that aim to abrogate these disrupted pathways. Several drugs are under development, but the only one with a proven survival benefit is sorafenib [13]. However, the adverse events were as high as 80% in sorafenib therapy, and treatment interruption owing to side effects was recorded in 38% of treated patients [14]. In order to improve therapeutic efficacy, it is urgent to find a new targeting therapy gene, reveal its mechanisms, and try to effectively improve HCC treatment.

Recently, accumulating research results have been showing that MTDH could also promote malignant tumor growth. In a MTDH transgenic mouse model with hepatocyte-specific expression of MTDH, such mice displayed profound resistance to chemotherapeutics and growth factor deprivation with activation of pro-survival signaling pathways [11]. It is considered that MTDH could enhance tumor growth in multiple ways. First, MTDH could promote angiogenesis through increasing expression of angiogenesis molecular markers including angiopoietin-1 and hypoxia-inducible factor 1-alpha [15]. MTDH also down-regulated many transcriptional inhibitor factors such as FOXO1 and FOXO3a and thus promoted malignant cancer cell proliferation [16]. By promoting Bcl-2 expression, MTDH significantly inhibited lung cancer cell apoptosis [17]. Our results showed that MTDH shRNA inhibited HepG2 cell viability and induced cell apoptosis *in vitro* and *in vivo*, and these results strongly suggest that MTDH could also promote HCC growth.

In normal tissue development, PTEN functions as part of the process of coordinating the differentiation and proliferation of cell types in time and space to form a functional organ [18]. However, PTEN is a well-known tumor suppressor gene, and loss of PTEN expression isassociated with aggressive behavior and poor prognosis in cancer [19]. PTEN-deleted mice easily developed malignant tumors [20]. FOXO1/3 and PTEN depletion in granulosa cells also promoted ovarian granulosa cell tumor development [21]. MTDH could regulate multiple signaling pathways, including NF-kappaB signaling. Also, suppressing NF-kappaB signaling could promote PTEN transcript thus increase its expression [22,23]. In our study, knockdown of MTDH effectively increased PTEN expression in HepG2 cells. This reason might be that MTDH shRNA could suppress NF-kappaB signaling in HepG2 cells. Increasing PTEN could inhibit AKT activation, and thus suppress HepG2 cell proliferation and inhibit apoptosis [24]. The function of p53 as a tumor suppressor has been attributed to its ability to promote cell death or permanently inhibit cell proliferation. The p53 tumor suppressor is the central component of a complex network of signaling pathways that protect organisms against the propagation of cells carrying oncogenic mutations [25]. *P53*^−/−^ myoblasts transformed by K-ras overexpression resulted in impaired terminal differentiation and rapid, reproducible tumor formation following orthotopic injection into syngeneic host hindlimbs in mice [26]. However, PTEN deficiency could effectively suppress the abundance of p53 [27]. In our study, the increasing p53 might be that MTDH shRNA could up-regulate PTEN in HepG2 cells. These results strongly suggested that therapy targeting MTDH potentially has great value and deserves further investigation.

In conclusion, our results showed that MTDH expression was significantly increased in HCC cancer tissues compared to matched non-cancerous liver tissues. Knockdown of MTDH expression could significantly inhibit HepG2 cell proliferation, colony formation, and induce cell apoptosis. Mechanism analysis showed that MTDH shRNA decreased phosphorylation of AKT and increased apoptosis-related protein PTEN and p53 expression in HepG2 cells. *In vivo* results also showed that MTDH shRNA could effectively inhibit HepG2 tumor growth. All these results suggested that MTDH might function as a tumor growth promoter in HCC, meaning it potentially has great value in targeted therapy in HCC treatment.

## 4. Experimental Section

### 4.1. Cell Culture

HepG2 human HCC cell line was purchased from the American Type Culture Collection (ATCC, Rockville, MD, USA). Cells were cultured in Dulbecco’s modified Eagle medium (DMEM, Invitrogen, Carlsbad, CA, USA) supplemented with 10% fetal bovine serum, 100 IU/mL penicillin, and 100 μg/mL streptomycin at 37 °C in a 5% CO_2_ incubator.

### 4.2. RT-PCR of MTDH

At 80% confluency, cells were dissociated with 0.25% trypsin (Invitrogen) and collected for reverse transcription polymerase chained reaction (RT-PCR). The total RNA was isolated using TRIzol reagent (Invitrogen). Primers of MTDH and GAPDH (internal control) were synthesized (Shengong Bio, Shanghai, China). The forward primer sequences for MTDH were: AAGAGGAAAACTG AGCCATCTG, and reverse: CGGCTAACATCCCACTGATAAT. The forward primer sequences for GAPDH were: AGAAGGCTGGGGCTCATTTG, and reverse: AGGGGCCATCCACAGTCTTC, respectively. PCR was performed in a DNA thermal cycler (Applied Biosystems, Carlsbad, CA, USA) in the following conditions: one cycle at 94 °C for 2 min; 26 cycles, at 94 °C for 30 s, at 62 °C for 30 s, and at 72 °C for 45 s; and one cycle at 72 °C for 10 min. PCR products were electrophoresed on 1.5% agarose gel containing 0.5 μg/mL ethidium bromide and visualized using an ultraviolet illuminator.

### 4.3. Tissue Samples

HCC tissues and matched non-cancerous hepatic tissues were collected at the Department of Hepatibiliary Surgery in the Second Chongqin Medical Hospital, Chongqin, China between June 2013 and October 2014. The corresponding paraneoplastic tissues were taken at least 2 cm apart from the cancerous node. The tissues were immediately frozen in liquid nitrogen after surgical removal and stored at −80 °C until use. All the recruited patients in this study had no history of preoperative chemotherapy or radiotherapy. HCC diagnosis was based on World Health Organization criteria [28]. Liver function was assessed using the Child-Pugh scoring system. In this study, written informed consent was obtained from all patients, and the Chinese Medical Association Society of Medicine’s Ethics Committee approved all aspects of this study in accordance with the Helsinki Declaration.

### 4.4. Western Blot Analysis

HepG2 cells were collected and lysed with radio immunoprecipitation assay buffer (50 mM Tris-HCl pH 7.4, 150 mM NaCl, 1% (*v*/*v*) NP40, 0.1% (*w*/*v*) SDS, 0.5% (*w*/*v*) sodium deoxycholate) with proteas inhibitors. Equal amounts of proteins (50 μg) were separated on 10% sodium dodecyl sulfate-polyacrylamide gel electrophoresis (SDS-PAGE) gels and then transferred to a polyvinylidene difluoride membrane (Millipore, Bedford, MA, USA). After blocking with 5% non-fat milk, the membrane was incubated overnight at 4 °C with the primary MTDH antibody (Abcam Inc., Cambridge, CA, USA; 1:10,000), GAPDH (Cell Signal, Boston, MA, USA; 1:1000) , PCNA (Cell Signal, 1:1000), p-AKT (Cell Signal, Boston, 1:1000), AKT (Cell Signal, 1:1000), p53 (Cell Signal, 1:1000), and PTEN (Cell Signal, 1:1000) then with horseradish peroxydase-coupled secondary antibody (Cell Signal, USA). Signal was detected with enhanced chemiluminescence (Millipore, Bedford, MA, USA). Band signals were acquired in the linear range of the scanner and analyzed using QUANTITY ONE software (Bio-Rad, Hercules, CA, USA).

### 4.5. siRNA-Mediated Silencing of MTDH

Lentiviruses were generated in 293T cells by co-transfection plasmid of pGCSIL-GFP-shRNA-MTDH or pGCSIL-GFP-shRNA-NC, with pHelper1.0 and pHelper2.0 plasmids at GeneChem Technology (Shanghai, China). The MTDH RNA interference lentivirus vector named LV-GFP-MTDH-shRNA and LV-GFP-NC-shRNA (control). The shRNA targeting sequencing for MTDH was: 5′-GAGUUGAUGAUCGUAGAAG-3′ (siMTDH) and negative control siRNA was 5′-UUCUCCGAACGUGUCACGU-3′ (siNC). The HepG2 cell line was infected with LV-GFP-MTDH-shRNA or LV-GFP-NC-shRNA for 2 h and subsequently placed in fresh medium, while the uninfected cells were used as the blank control. The cells were cultured for the next 48 h and then harvested for western blot analysis or prepared for the following experiments.

### 4.6. CCK-8 Assay

Cell Counting Kit-8 (Dojindo, Kumamoto, Japan) was used to measure the tumor cell proliferation. Cells were plated at a density of 5000 cells per well in 96-well plates with the complete medium. At the end of infection LV-GFP-MTDH-shRNA or LV-GFP-NC-shRNA for 48 h, 10 μL of the cell proliferation reagent WST-8 was added to each well and incubated for 2 h at 37 °C. The absorbance was measured at 450 nm with an ultraviolet spectrometer (Beckman Coulter, Brea, CA, USA). The experiments were performed in quadruplicate and repeated in triplicate.

### 4.7. Colony Formation Assay

After the infection LV-GFP-MTDH-shRNA or LV-GFP-NC-shRNA for 48 h in HepG2 cells, 500 cells were plated into 6-well plates and continually cultured at 37 °C and at an atmosphere of 5% CO_2_ for 10 days. The supernatants were then discarded and cells were rinsed in PBS for twice, and fixed with 70% ethanol for 15 min. The cells were stained with 0.1% crystal violet for 10 min and PBS was used to wash the rest of crystal violet twice. The plates were dried at room temperature and the colony numbers containing more than 50 cells were microscopically counted. The experiments were performed in triplicate.

### 4.8. Cell Apoptosis Assay

HepG2 cells were infected with LV-GFP-MTDH-shRNA or LV-GFP-NC-shRNA for 48 h, and an apoptosis assay was performed with Alexa Fluor 488 annexin V/Dead Cell Apoptosis Kit (Invitrogen) in 48 h after infection according to the manufacturer’s instructions. The cell suspension (100 μL) was incubated with 5 μL of annexin-V and 1 μL of propidium iodide at room temperature for 15 min. The stained cells were analyzed with fluorescent-activated cell sorting (FACS) using BD LSR II flow cytometer (BD Biosciences, San Diego, CA, USA). The percentage of early apoptotic cells located in the lower right quadrant (annexin V–FITC positive/PI negative cells), as well as late apoptotic cells located in the upper right quadrant (annexin V–FITC positive/PI positive cells) were determined.

### 4.9. Immunohistochemical Analysis

MTDH expression was detected in 4-μm thick HCC paraffin embedded slides with an immunohistochemical staining method. Briefly, the slides were first dewaxed in xylol and rehydrated in 100%, 95%, and 85% graded alcohol series, with antigen retrieval in 0.01 M sodium citrate solution 98 °C for 15 min. Endogenous peroxidase activity was blocked with 3% H_2_O_2_-methanol and normal goat serum was used to close non-specific binding. An anti-human MTDH rabbit monoclonal antibodies (Abcam Inc., Cambridge, CA, USA; 1:100) or PCNA (Cell Signaling, Boston, MA, USA; 1:100) was incubated at 4 °C overnight. The next morning, the slides were washed three times with PBS and incubated with a biotin-labeled second antibody for 15 min. DAB color-substrate solution was added for half a minute. Then the slides were counterstained with hematoxylin for 1 min, dried in 60 °C for 2 h, dehydrated in 85%, 95%, and 100% gradient ethanol, and sealed with neutral gum. The slides omitted the first antibody used as negative controls, and breast cancer tissue slides which had been confirmed to overexpress the MTDH protein as positive controls.

### 4.10. Immunohistochemical Results Evaluation

A semi-quantitative assessment method which combining staining intensity and the percentage of positive cells was used to evaluate MTDH dyeing results. The staining intensity was scored as 0, negative; 1, weak; 2, moderate; and 3, strong. The percentage of positive cells was scored as (0, <21%; 1, 21% to 40%; 2, 41% to 60%; 3, 61% to 80%; and 4, 81% to 100%). The final staining score was calculated by a staining index (SI) of MTDH ranging from 0 to 12 that was finally determined by multiplying the scores of staining intensity and proportion of immunopositive cancer cells according to previously published criteria [9]. A total score less than 6 was considered low MTDH expression, while a score equal to or greater than 6 indicated high MTDH expression. All the sections were independently assessed by two experienced pathologists, and inconsistent immunohistochemical results were reviewed again by the two pathologists and to obtain the final pathological diagnosis.

### 4.11. In Vivo Tumorigenicity

For the HCC xenograft model, 4-week-old BALB/c nude mice were housed in a pathogen-free environment and used for experimentation. Medium (0.2 mL) containing 5 × 10^6^ HepG2 cells at the end of LV-GFP-MTDH-shRNA or LV-GFP-NC-shRNA infection for 48 h was injected subcutaneously into the left posterior flank regions of each mouse. Tumor growth was examined every week. Mice were sacrificed after tumor inoculation for six weeks, and the volume of each tumor was calculated. Tumor volume was calculated using the formula LS/2, where L is the greatest tumor diameter and S is the lowest tumor diameter. All experimental procedures were conducted in accordance with the Guide for the Care and Use of Laboratory Animals and approved by our institutional ethical guidelines for animal experiments.

### 4.12. Statistical Analysis

All the data were analyzed with SPSS 17.0 software (SPSS, Chicago, IL, USA). Measurement data were presented as means ± SD from at least three separate experiments. A two-sided Student’s *t*-test was used to analyze the differences in MTDH expression and HepG2 cell proliferation, colony formation number, and apoptotic rate. Statistical significance was considered to be *p <* 0.05.

## 5. Conclusions

In conclusion, we found that MTDH high expression in HCC tissues and higher MTDH protein levels are found in most HCC cancerous tissues compared with their matched adjacent non-tumor tissues. Additionally, the MTDH mRNA was also higher in HCC tissues compared to their matched adjacent non-tumor tissues. Knockdown of the endogenous MTDH using small interfering RNA further showed that deficiency of MTDH suppressed cell growth and caused apoptosis in HCC cells. And mechanisms analysis showed that knockdown MTDH expression promoted PTEN and p53 expression in HCC cells. And phosphorylation of AKT was inhibited by MTDH siRNA. Knockdown MTDH also inhibited tumor growth in vivo. All these results indicated that MTDH protein levels in most HCC tissues are higher than non-tumor tissues, and knockdown of MTDH inhibited growth and induced apoptosis in HCC cells through the activation of PTEN. Therefore, MTDH might be an effective targeted therapy gene for HCC.
